# RFX1 participates in doxorubicin‐induced hepatitis B virus reactivation

**DOI:** 10.1002/cam4.1468

**Published:** 2018-03-30

**Authors:** Jie Wang, Junqiao Jia, Ran Chen, Shanlong Ding, Qiang Xu, Ting Zhang, Xiangmei Chen, Shuang Liu, Fengmin Lu

**Affiliations:** ^1^ State Key Laboratory of Natural and Biomimetic Drugs Department of Microbiology & Infectious Disease Center School of Basic Medical Sciences Peking University Health Science Center Beijing China; ^2^ Beijing Artificial Liver Treatment & Training Center Beijing Youan Hospital Capital Medical University Beijing China

**Keywords:** Doxorubicin, epirubicin, hepatitis B virus, reactivation, regulatory factor X box 1

## Abstract

Cytotoxic chemotherapy drugs, including doxorubicin, can directly promote hepatitis B virus (HBV) replication, but the mechanism has not been fully clarified. This study investigated the potential mechanism underlying the cytotoxic chemotherapy‐mediated direct promotion of HBV replication. We found that HBV replication and *regulatory factor X box 1* gene (RFX1) expression were simultaneously promoted by doxorubicin treatment. The amount of RFX1 bound to the HBV enhancer I was significantly increased under doxorubicin treatment. Furthermore, the activity of doxorubicin in promoting HBV replication was significantly attenuated when the expression of endogenous RFX1 was knocked down, and the EP element of HBV enhancer I, an element that mediated the binding of RFX1 and HBV enhancer I, was mutated. In addition, two different sequences of the conserved EP element were found among HBV genotypes A‐D, and doxorubicin could promote the replication of HBV harboring either of the conserved EP elements. Here, a novel pathway in which doxorubicin promoted HBV replication via RFX1 was identified, and it might participate in doxorubicin‐induced HBV reactivation. These findings would be helpful in preventing HBV reactivation during anticancer chemotherapy.

## Introduction

Cancer is the leading cause of human death globally, and nearly one of every six deaths is due to cancer. Human malignancies are responsible for 8.8 million deaths in 2015, and the number of new cases is predicted to rise by approximately 70% over the next two decades 1. In addition to surgery and radiotherapy, chemotherapy remains the most common treatment for cancer.

Hepatitis B virus (HBV) reactivation has been confirmed to frequently occur in hepatitis B surface antigen (HBsAg)‐positive HBV‐infected individuals who receive cancer chemotherapy 2, 3, 4, 5, 6, 7, 8, 9. HBV reactivation may also occur in previously resolved or inactive HBV carriers, but the risk seems to be lower 4, 10, 11, 12. It is estimated that almost 2 billion people worldwide, nearly one‐third of the world's population, have been infected with HBV. With increasing life expectancy and the expected global increase in new cancer cases, chemotherapy‐induced HBV reactivation is likely to become a more serious public health problem 13. The clinical manifestations of HBV reactivation range from asymptomatic, self‐limiting hepatitis to severe liver failure. Impaired liver function due to HBV reactivation may also lead to a delay or the interruption of the chemotherapy regimen, which is likely to increase the risk of morbidity and mortality associated with the underlying malignancy 14.

HBV reactivation should be defined as a marked increase in HBV replication (≥2 log increase from baseline levels or a new appearance of HBV DNA to a level of ≥100 IU/mL) in a person with previously stable or undetectable levels 15, 16. The types of HBV reactivation are described as follows: exacerbation of chronic hepatitis B, that is, a relative or absolute increase in the HBV DNA levels compared with the baseline levels, and a reactivation of past hepatitis B, that is, a reemergence of HBsAg in HBsAg‐negative/anti‐HBc‐positive patients 17. Chemotherapy is associated with a risk of HBV reactivation or exacerbation in 38% to 78% of HBV carriers 18, 19.

The causative factor of HBV reactivation has been mainly considered to be the loss of immune control over viral replication. During chemotherapy, when the immune system is suppressed, HBV replication is activated dramatically, and increased viral load may cause the widespread infection of hepatocytes. Once chemotherapy is stopped, immune function will be restored. Liver cells containing HBV may trigger strong immune‐mediated reactions and subsequently lead to liver damage 13, 20, 21, 22, 23, 24. Anthracyclines, such as doxorubicin or daunorubicin, are commonly used cytotoxic antibiotics in the chemotherapy of anticancer therapy, which are listed in the World Health Organization (WHO) model list of essential medicines 25, 26. HBV reactivation is more likely to occur in patients who receive cytotoxic chemotherapy as part of their chemotherapeutic regimen 27, 28, 29. For cytotoxic chemotherapy, other than its immunosuppressive effect, the mechanisms by which HBV is reactivated or exacerbated are still not fully clarified. It has been reported that promyelocytic leukemia protein (PML) and its associated PML nuclear body (PML‐NB) link the DNA damage response to HBV replication 30. HBV‐core protein (HBc) and HBV covalently closed circular DNA (cccDNA) are located in PML‐NB where they are associated with PML and histone deacetylase 1 (HDAC1). During cytotoxic chemotherapy, the enhanced binding of PML and HBc decreased the activity of PML‐associated HDAC1 and promoted the activity of the basal core promoter, which resulted in the promotion of pregenomic RNA (pgRNA) expression and the subsequent productions of HBc and HBV DNA but not of HBsAg and hepatitis B surface antigen (HBeAg). Accordingly, under short‐term cytotoxic chemotherapy, the production of HBsAg and HBeAg would not be promoted by cytotoxic chemotherapy‐induced DNA repair signaling. However, it has been reported that, except for HBc and HBV DNA, cytotoxic chemotherapy could promote the expressions of HBsAg and HBeAg under short‐term cytotoxic chemotherapy, including doxorubicin and epirubicin 31, 32. Therefore, except for PML and PML‐NB, another pathway may be involved in the promotion of HBV replication induced by cytotoxic chemotherapy. It has been reported that *regulatory factor X box 1* gene (RFX1), the mammalian homologue of the *CRT1* gene, is transcriptionally activated in response to DNA damage 33. Moreover, RFX1 can directly promote HBV replication 34, 35. Therefore, in this study, the role of RFX1 in the cytotoxic chemotherapy‐mediated promotion of HBV replication was investigated.

## Materials and Methods

### Plasmids

The 1.2 × HBV construct (pBB4.5‐HBV1.2, genotype C) was constructed using a 1.2‐fold length genome of genotype C HBV 36, based on pBB4.5‐HBV1.3 (genotype D, G1896A mutation), which was kindly provided by Professor Stephen A. Locarnini from the Victorian Infectious Diseases Reference Laboratory, Australia 37. The HBV expression vectors, pGEM‐HBV1.3A (genotype A) and pGEM‐HBV1.3B (genotype B), were kindly provided by Professor Ningshao Xia from the School of Public Health, Xiamen University, China. The 1.2 × HBV EP element mutant plasmids pBB4.5‐HBV1.2 EPM1 and pBB4.5‐HBV1.2 EPM2 were generated from the pBB4.5‐HBV1.2 plasmid using a QuikChange site‐directed mutagenesis kit (Stratagene, La Jolla, CA). The pCMV‐HA‐RFX1 plasmid was constructed by reverse transcription–polymerase chain reaction (RT‐PCR) of the mRNA extracted from HepG2, and the PCR products were subsequently ligated to the pCMV‐HA plasmid after digestion with EcoRI and NotI. The pGL3‐HBV EnhI‐luciferase plasmid was constructed by PCR of the pBB4.5‐HBV1.2 plasmid, and the PCR product, including the sequences of HBV enhancer I and the X promoter, was subsequently ligated into the pGL3‐basic plasmid after digestion with KpnI and XhoI. The pGL3‐HBV EnhI (EPM1)‐luciferase and pGL3‐HBV EnhI (EPM2)‐luciferase plasmids were generated from the pGL3‐HBV EnhI‐luciferase plasmid using QuikChange site‐directed mutagenesis kit (Stratagene). The pRNA‐U6.1‐RFX1 shRNA and pRNA‐U6.1‐scramble shRNA plasmids were constructed by annealing two oligonucleotides and subsequently ligated into the pRNA‐U6.1/Neo vector after digestion with BamHI and HindIII. The primer sequences used for plasmid construction are listed in Table S1.

### Cell transfection

Human hepatocellular carcinoma cell lines HepG2 and HuH7 were maintained in Dulbecco's modified Eagle medium supplemented with 10% fetal bovine serum (Gibco, Carlsbad, CA). The HBV stable expression cell lines HepG2.2.15 38 and HepAD38 39 were maintained in Dulbecco's modified Eagle medium (CellGro, Herndon, VA) supplemented with 10% fetal bovine serum (Gibco) and 400 *μ*g/mL G418 (Merck, Kenilworth, NJ). HepAD38 cells were also supplemented with 2 *μ*g/mL doxycycline (Merck) to block HBV replication, and doxycycline was removed for at least 1 week to produce HBV. Primary human hepatocytes (PHHs) were kindly provided by Professor Hongkui Deng from the Stem Cell Research Center and the Department of Cell Biology, Peking University. PHH cells were maintained in primary hepatocyte maintenance medium (PMM). HepG2 and HuH7 cells were seeded in a 12‐well plate at 4 × 10^5^ and 2 × 10^5^ cells/well, and in a 35 mm dish at 8 × 10^5^ and 4 × 10^5^ cells/well, respectively. Following the manufacturer's protocol, the cells were transfected with plasmids using lipofectamine 2000 (Life Technologies, Carlsbad, CA).

### Cell counting kit‐8 (CCK8) assay

HepG2 cells were seeded in 96‐well plates at a density of 2 × 10^4^ cells per well. After 24 h of doxorubicin treatment, CCK‐8 (Dojindo Laboratories, Rockville, MA) reagent (10 *μ*L/well) was added and incubated for 1 h. Following vortexing for 5 min, the absorbance value of each well was measured at 450 nm. Each sample was tested in six repeats in three separate experiments.

### Dual‐luciferase reporter assay

HepG2 and HuH7 cells were transiently cotransfected with pGL3‐HBV Enh I (wild‐type, EPM1, or EPM2)‐luciferase, TRL‐PK plasmid, and pCMV‐HA‐RFX1 expression plasmid or vector control pCMV‐HA using lipofectamine 2000 in a 12‐well plate. The luciferase activity in each well was quantified using a dual‐luciferase reporter assay kit (Promega, Madison, WI) at 36 h post‐transfection and was detected by an EnSpire multimode plate reader (PerkinElmer, Waltham, MA) following the manufacturer's protocol.

### Quantitative reverse transcription‐PCR

Total RNA from HepG2.2.15, HepAD38, HepG2 or HuH7 cells was extracted with TRIzol reagent (Invitrogen, Carlsbad, CA), according to the manufacturer's protocol, and treated with DNase I (Thermo Fisher Scientific, Waltham, MA). The isolated RNA was reverse‐transcribed using RevertAid First Strand DNA Synthesis Kit (Thermo Fisher Scientific). The primers used for qRT‐PCR are shown in Table S2.

The reaction was carried out in a MicroAmp Optical 96‐well plate using SYBR Green PCR Master Mix (Roche, Mannheim, Germany), with 1 *μ*L cDNA in each well. PCRs were monitored in real‐time using the Roche Lightcyler 480II Real‐time PCR System (Roche). The procedures of the PCR were 95°C for 5 min; 95°C for 20 sec, 60°C for 20 sec, and 72°C for 20 sec, 40 cycles; and 72°C for 7 min. The mRNA of the *ACTB* (*β*‐actin) gene was used as the internal control.

### Quantification of HBV DNA

The levels of HBV DNA were detected by an HBV DNA Quantitative Detection (TaqMan) Kit (Sansure Biotech, Changsha, Hunan, China) according to the manufacturer's instruction.

### HBV infection

HBV infection of PHH was conducted as previously described 40. Briefly, 1 × 10^5^ PHH cells in a 48‐well plate were inoculated with 1 × 10^7^ copies of the genome equivalent of HBV in the presence of 4% PEG8000 for 20 h. PHH cells were then washed with PBS six times and maintained in PMM medium with medium change every 2–3 days.

### Detection of HBsAg and HBeAg

The levels of HBsAg and HBeAg were measured by a time‐resolved fluoroimmunoassay (TRFIA) as previously described 41, 42. In brief, the culture supernatant (100 *μ*L) was added to a microtiter plate coated with anti‐HBs or anti‐HBe, shaken for 40 min at room temperature, and washed four times. Europium‐labeled anti‐HBsAg or anti‐HBeAg were diluted 1:50 by HBsAg or HBeAg dilution buffer and added at 100 *μ*L per well, shaken for 40 min at room temperature, and washed six times. At last, after incubation with enhancement solution (100 *μ*L) for 5 min, the plates were read using an Anytest reader (PerkinElmer).

### Western blot

The protein lysates (total 60 *μ*g) were dissolved in 1 × laemmli buffer (with 5% 2‐mercaptoethanol), boiled for 10 min, and resolved on a 4–12% gradient SDS‐PAGE gel (Bio‐Rad, Hercules, CA, USA). Subsequently, the proteins were transferred to a nitrocellulose membrane and incubated with mouse anticore protein antibody, mouse anti‐HBx antibody (the above two antibodies were both gifted from State Key Laboratory of Molecular Vaccinology and Molecular Diagnostics, School of Public Health, Xiamen University. Xiamen, Fujian, China), mouse anti‐p53 (Human) antibody (MBL International, Woburn, MA), rabbit anti‐HA‐tag antibody (MBL International), rabbit anti‐RFX1 antibody (Gene Tex, Irvine, CA), or rabbit anti‐*α*‐tubulin antibody (MBL International) overnight at 4°C. Reactive proteins were developed with anti‐mouse and anti‐rabbit antibodies conjugated to horseradish peroxidase (Zhongshan Golden Bridge, Beijing, China) and visualized using SuperSignal^™^ West Femto Maximum Sensitivity Substrate (Thermo Fisher Scientific) according to the manufacturer's protocol.

### Chromatin immunoprecipitation

The chromatin immunoprecipitation (ChIP) assay was performed as described previously 43. The cell lysates were incubated with rabbit anti‐RFX1 antibody (Gene Tex) or IgG from rabbit serum (Sigma‐Aldrich, St Louis, MO).

### Statistical analysis

The Student's *t*‐tests were performed using the statistical software package SPSS version 21.0 for Windows (SPSS, Chicago, IL). All tests of significance were two‐tailed, and *P *<* *0.05 was considered statistically significant. Data were shown as the mean ± SD of at least three independent experiments, * indicated *P *<* *0.05, ** indicated *P *<* *0.01, and *** indicated *P *<* *0.001.

## Results

### Doxorubicin promotes HBV replication

**Figure 1:**
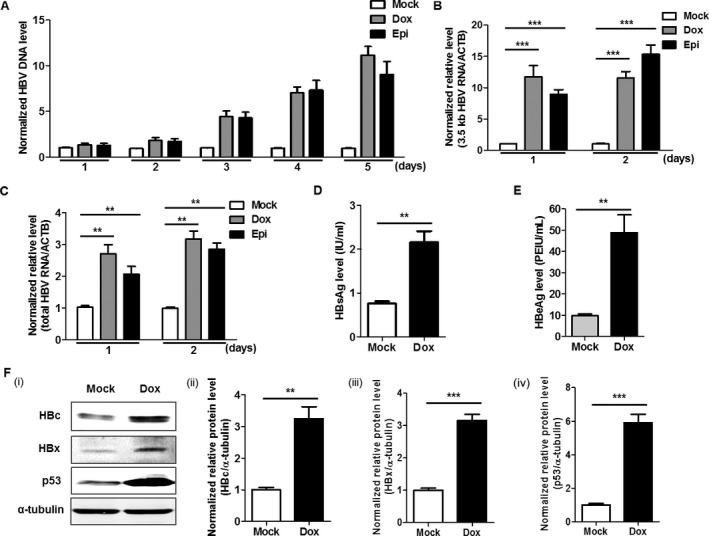
Doxorubicin and epirubicin promote HBV replication in HepG2.2.15 cells. (A) HepG2.2.15 cells were treated with either 1 *μ*mol/L doxorubicin (Dox) or epirubicin (Epi) for 1 h. The normalized levels of HBV DNA in the culture supernatant at different times (1, 2, 3, 4, and 5 days) post‐treatment were measured by quantitative PCR (qPCR). HepG2.2.15 cells were treated with either 1 *μ*mol/L Dox or Epi for 1 h, 24, or 48 h later, and the normalized relative levels of 3.5 kb (B) and total HBV RNA (C) in HepG2.2.15 cells were quantified by quantitative RT‐PCR (qRT‐PCR). The mRNA of the *β‐actin* gene (ACTB) was used as an internal control. The levels of HBsAg (D) and HBeAg (E) in the culture supernatant of HepG2.2.15 cells treated with 1 *μ*mol/L Dox for 1 h were measured by a time‐resolved fluoroimmunoassay at 48 h post‐Dox treatment. (F) The protein levels of HBc (HBc) and HBxAg (HBx) in HepG2.2.15 cells treated with 1 *μ*mol/L Dox for 1 h were measured by Western blot at 48 h post‐Dox treatment. (i). The right figures were the statistical graphs of the HBc (ii), HBx (iii), and p53 (iv) protein levels detected by Western blot, which was analyzed by ImageJ software (NIH, Bethesda, MD), at least in triplicate. The p53 protein was used as a positive control. The *α*‐tubulin was used as an internal control.

HepG2.2.15 cells, a commonly used HBV replication cell model, were treated with 1 *μ*mol/L doxorubicin or its derivative, epirubicin. As shown in Figure 1A, compared o the levels of HBV DNA in the vehicle (DMSO) control (which was standardized as 1), gradually increasing HBV DNA levels in the culture supernatants of HepG2.2.15 cells were observed from day 3 to day 5 following doxorubicin or epirubicin treatment. As the expression of HBV RNA in the cells occurs earlier than does the formation of HBV DNA viral particles in the culture supernatant, the changes in the HBV RNA levels should be earlier than those of the HBV DNA. To test the effect of doxorubicin and epirubicin on the expression of HBV RNA, the levels of 3.5 kb HBV RNA and total HBV RNA were analyzed. As shown in Table S2, the 3.5 kb HBV RNA levels were detected by the primers near the 5′ terminus of 3.5 kb HBV RNA, and the total HBV RNA levels were detected by the primers near the 3′ terminus of all the HBV RNAs. The measurement of 3.5 kb HBV RNA reflects the expressions of the pre‐C mRNA and pgRNA, which are regulated by the core promoter/basic core promoter. As the expressions of 2.4 kb HBV RNA and 2.1 kb HBV RNA are much more than that of the 3.5 kb HBV RNA and 0.7 kb HBV RNA, the measurement of the total HBV RNA reflects the expressions of 2.4 kb HBV RNA and 2.1 kb HBV mRNA, which is regulated by the SP1 and SP2 promoters, respectively. Therefore, the measurement of 3.5 kb HBV RNA and total HBV RNA could reflect the effect of doxorubicin on the regulation of different HBV promoters. The results revealed that the levels of 3.5 kb HBV RNA and the total HBV RNA in HepG2.2.15 cells both significantly increased at 1 and 2 days following doxorubicin or epirubicin treatment (Fig. 1B and C). The levels of HBsAg and HBeAg in the culture supernatant of HepG2.2.15 cells were also significantly increased after doxorubicin treatment (Fig. 1D and E). Furthermore, using Western blot analysis, the levels of HBc and HBV × proteins (HBx) were significantly increased in doxorubicin‐treated HepG2.2.15 cells, which were relatively quantified by gray scale analysis (Fig. 1Fi, Fii and Fiii). Additionally, the increased p53 protein expression demonstrated the cytotoxic effect of doxorubicin (Fig. 1Fi and Fiv). Consistently, the viral load of HBV in the culture supernatant of HepAD38 cells, which is another commonly used HBV replication cell model, was also significantly promoted by doxorubicin (Fig. S1).

### Doxorubicin promotes the expression of RFX1 gene

**Figure 2:**
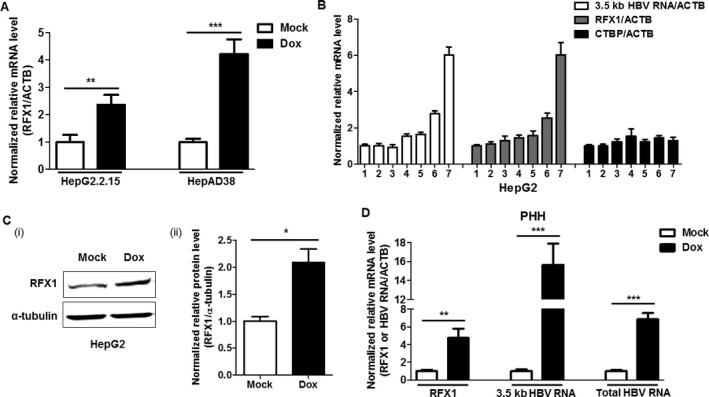
Doxorubicin promotes RFX1 expression and HBV replication in parallel. (A) HepG2.2.15 and HepAD38 cells were treated with 1 *μ*mol/L Dox for 1 h, respectively. The mRNA levels of RFX1 in each cell were quantified by qRT‐PCR at 48 h post‐Dox treatment. (B) The pBB4.5‐1.2 × HBV expression plasmid (2 *μ*g) was transfected into HepG2 cells using lipofectamine 2000 in a 35‐mm dish. Then, the transfected HepG2 cells were treated with serial dosages of Dox for 1 h. The relative levels of 3.5 kb HBV RNA, RFX1, and CTBP mRNA in the cells were quantified by qRT‐PCR at 48 h post‐Dox treatment. ACTB mRNA was used as an internal control. The concentrations of Dox were 1: 0 *μ*mol/L, 2: 0.05 *μ*mol/L, 3: 0.1 *μ*mol/L, 4: 0.2 *μ*mol/L, 5: 0.5 *μ*mol/L, 6: 1 *μ*mol/L, and 7: 2 *μ*mol/L. (C) The levels of RFX1 protein in HepG2 cells treated with or without 1 *μ*mol/L Dox for 1 h were detected by Western blot at 48 h post‐Dox treatment (i). The right figure (ii) was the statistical graph of RFX1 protein levels detected by Western blot, which were analyzed by ImageJ software (NIH), at least in triplicate. The *α*‐tubulin was used as an internal control. (D) 1 × 10^5^ primary human hepatocytes (PHH) cells in a 48‐well plate were inoculated with 1 × 10^7^ copies of genome equivalent HBV in the presence of 4% PEG 8000 for 20 h. PHH cells were then washed with phosphate‐buffered solution (PBS) six times and maintained in PMM medium for 3 days. Then, the HBV‐infected PHH cells were treated with 1 *μ*mol/L Dox for 1 h. The relative levels of 3.5 kb HBV RNA, total HBV RNA, and RFX1 mRNA in the cells were quantified by qRT‐PCR at 48 h post‐Dox treatment. ACTB mRNA was used as an internal control.

As the expression of RFX1, a transactivator of HBV replication, can be promoted in the context of DNA damage, it is possible that RFX1 may participate in the direct promotion of HBV replication induced by cytotoxic chemotherapy. To test this possibility, the effect of doxorubicin on RFX1 expression was analyzed. In this study, we found that RFX1 expression was also upregulated after doxorubicin treatment (Fig. 2A). Further, as shown in Figure 2B, the levels of HBV RNAs (3.5 kb HBV RNA and total HBV RNA) and RFX1 mRNA gradually increased in a dose‐dependent manner when HepG2 cells were transfected with 1.2 × HBV expression plasmids and treated with a serial dosage (0, 0.05, 0.1, 0.2, 0.5, 1, and 2 *μ*mol/L in final concentrations) of doxorubicin. To test the specificity and reliability of the effect of doxorubicin on HBV replication and RFX1 expression, the mRNA levels of the *C‐terminal binding protein* gene (CTBP), a relatively stable gene in HepG2 cells 44, were analyzed in parallel. The results revealed that CTBP expression was still stable following doxorubicin treatment, suggesting that doxorubicin could specifically promote HBV replication and RFX1 expression (Fig. 2B). The RFX1 protein levels were also significantly increased in doxorubicin‐treated HepG2 cells (Fig. 2Ci and Cii). In line with this finding, doxorubicin could dose‐dependently promote HBV replication and RFX1 expression in HuH7 cells (Fig. S2). In addition, to further demonstrate this phenomenon, primary human hepatocyte (PHH) cells were inoculated with HBV collected from the culture supernatant of HepAD38 cells, and the levels of 3.5 kb HBV RNA, total HBV RNA, and RFX1 mRNA in the PHH cells were quantified by qRT‐PCR. Consistently, in PHH cells, HBV replication and RFX1 expression were both significantly promoted by doxorubicin (Figs. 2D and S3).

### RFX1 participates in the direct promotion of HBV replication mediated by doxorubicin

**Figure 3:**
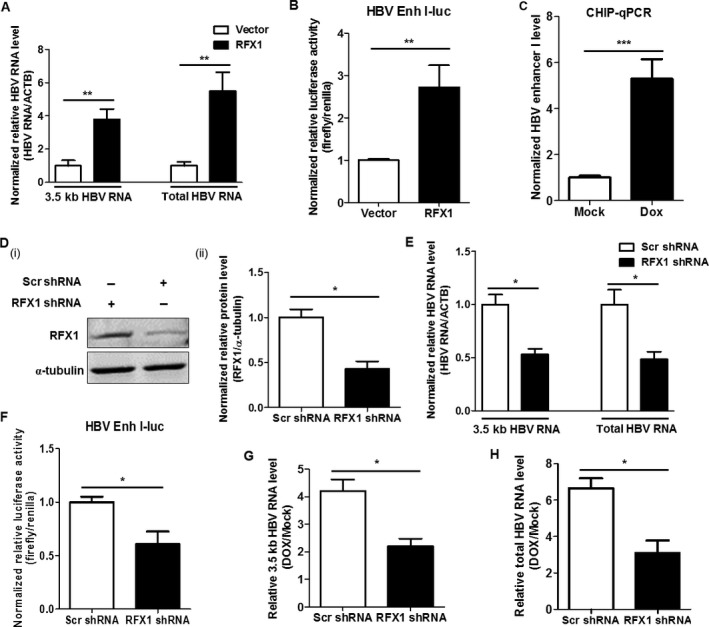
RFX1 promotes HBV replication by enhancing the activity of HBV enhancer I. (A) HepG2 cells were cotransfected with pBB4.5‐1.2 × HBV expression plasmid (1 *μ*g) and pCMV‐HA‐RFX1 expression plasmid or vector control pCMV‐HA (1 *μ*g) using lipofectamine 2000 in a 35‐mm dish. The relative levels of 3.5 kb HBV RNA and total HBV RNA in the cells were quantified by qRT‐PCR. ACTB mRNA was used as an internal control. (B) HepG2 cells were cotransfected with pGL3‐HBV Enh I‐luciferase (Firefly), TRL‐PK (Renilla), and pCMV‐HA‐RFX1 expression plasmids or vector control pCMV‐HA using lipofectamine 2000 in a 12‐well plate. The effect of RFX1 in the activity of HBV enhancer I was detected by a dual‐luciferase assay. (C) HepAD38 cells were treated with 1 *μ*mol/L Dox for 1 h, and the effect of Dox in the binding activity of RFX1 to HBV enhancer I (Enh I) was analyzed by ChIP‐PCR at 24 h post‐Dox treatment. (D) The levels of RFX1 protein in the pooled HepG2 cells stably transfected with RFX1 shRNA or scramble shRNA expression plasmid were detected by Western blot (i). The statistical graph (ii) of RFX1 protein levels detected by Western blot was analyzed by Image J software (NIH) at least in triplicate. The *α*‐tubulin was used as an internal control. (E) HepG2‐Scr and HepG2‐RFX1 shRNA cells were transfected with a pBB4.5‐1.2 × HBV expression plasmid (2 *μ*g) using lipofectamine 2000 in a 35‐mm dish. The levels of 3.5 kb HBV RNA, total HBV RNA, and ACTB mRNA in cells were quantified by qRT‐PCR. ACTB mRNA was used as an internal control. (F) HepG2‐Scr and HepG2‐RFX1 shRNA cells were cotransfected with pGL3‐HBV Enh I‐luciferase and TRL‐PK using lipofectamine 2000 in a 12‐well plate. The effect of RFX1 in the activity of HBV enhancer I was detected by a dual‐luciferase assay. HepG2‐Scr and HepG2‐RFX1 shRNA cells were transfected with a pBB4.5‐1.2 × HBV expression plasmid (2 *μ*g) using lipofectamine 2000 in a 35‐mm dish. After 24 h of transfection, the cells were treated with 1 *μ*mol/L Dox for 1 h. The relative levels of 3.5 kb HBV RNA (G) and the total HBV RNA (H) in cells were quantified by qRT‐PCR at 48 h post‐Dox treatment. ACTB mRNA was used as an internal control.

It has been reported that RFX1 can promote HBV replication by enhancing HBV enhancer I 34, 35. To further demonstrate the effect of RFX1 in HBV replication, the levels of 3.5 kb HBV RNA and total HBV RNA were detected by qRT‐PCR. In line with previous reports, RFX1 could significantly upregulate the levels of 3.5 kb HBV RNA and total HBV RNA (Fig. 3A). To further demonstrate that RFX1 can enhance the activity of HBV enhancer I, the sequence of HBV enhancer I and the X promoter was constructed into the luciferase reporter assay system, and the effect of RFX1 on HBV enhancer I was evaluated by a dual‐luciferase assay. As shown in Figure 3B, the overexpression of RFX1 indeed could promote the activity of HBV enhancer I. The ectopic expression of RFX1 was confirmed by Western blot (Fig. S4). In addition, the binding activity of RFX1 to the HBV enhancer I was analyzed by ChIP‐PCR. The results showed that the amount of RFX1 bound to HBV enhancer I was significantly increased under doxorubicin treatment (Fig. 3C).

Next, the endogenous RFX1 of HepG2 cells were stably knocked down by RFX1‐specific shRNA (Fig. 3D). Compared to the control HepG2‐Scr cells, which were stably transfected with scramble shRNA, HBV replication was significantly downregulated in RFX1 stable knockdown HepG2‐RFX1 shRNA cells transfected with 1.2 × HBV expression plasmid (Fig. 3E). Meanwhile, the activity of HBV enhancer I decreased when endogenous RFX1 was knocked down (Fig. 3F). Moreover, compared to HepG2‐Scr cells, the extent of doxorubicin‐mediated promotion on the 3.5 kb HBV RNA level was significantly reduced when the 1.2 × HBV plasmid‐transfected HepG2‐RFX1 shRNA cells were treated with doxorubicin (Fig. 3G). Consistently, the doxorubicin‐mediated promotion of the total HBV RNA level was also significantly reduced when RFX1 was knocked down (Fig. 3H). To exclude the cytotoxic effect of RFX1 in HBV replication during doxorubicin treatment, a CCK8 assay was performed in RFX1‐overexpressed HepG2 cells that were treated with serial dosages of doxorubicin. The result revealed that although doxorubicin has a dose‐dependent effect on the viability of cultured cells, there was no cytotoxic effect of RFX1 in HepG2 cells during doxorubicin treatment (Fig. S5).

### RFX1 mediates active HBV replication in an EP element‐dependent manner

**Figure 4:**
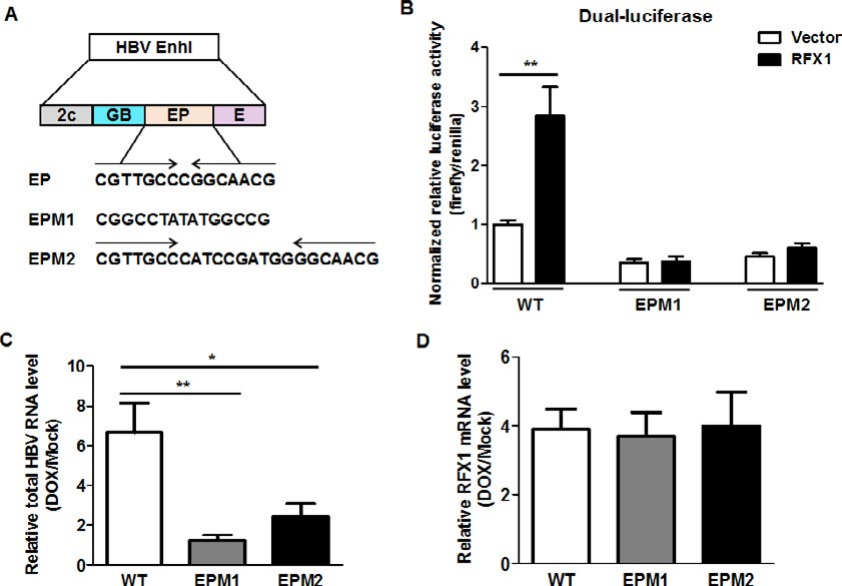
Doxorubicin promotes HBV replication depending on the EP element of HBV enhancer I. (A) The sequences of wild‐type, EPM1‐, and EPM2‐mutated EP elements. Enh I: enhancer I; 2c, GB, EP, and E are the HNF3, HNF4/RXR, RFX1/MIBP1, and basic leucine zipper‐binding sites, respectively. (B) HepG2 cells were cotransfected with pGL3‐HBV Enh I (wild‐type, EPM1 or EPM2)‐luciferase, TRL‐PK and pCMV‐HA‐RFX1 expression plasmids, or vector control pCMV‐HA using lipofectamine 2000 in a 12‐well plate. The role of the EP element in RFX1‐mediated activation of the HBV enhancer I was evaluated by a dual‐luciferase assay. HepG2 cells were transfected with wild‐type, EPM1, or EPM2 mutant 1.2 × HBV expression plasmid. After 24 h of transfection, the cells were treated with 1 *μ*mol/L Dox for 1 h. The levels of total HBV RNA (C) and RFX1 mRNA (D) were detected by qRT‐PCR at 48 h post‐Dox treatment.

It has been reported that RFX1 enhances the activity of HBV enhancer I via its binding to the EP element located in the HBV enhancer I 45, 46. Accordingly, EPM1 (CGGCCTATATGGCCG) and EPM2 (CGTTGCCCATCCGATGGGGCAACG), the mutants of the EP element, were introduced in the luciferase reporter assay system, respectively (Fig. 4A). Through the dual‐luciferase assay, we found that the RFX1‐mediated promotion of HBV enhancer I activity disappeared when the EP element was mutated (Fig. 4B).

To further investigate the participation of RFX1 in the doxorubicin‐mediated promotion of HBV replication, two EP element‐mutated 1.2 × HBV expression plasmids were constructed. Compared to the wild‐type HBV, the enhancement of HBV replication induced by doxorubicin was significantly reduced in HepG2 cells transfected with 1.2 × HBV expression plasmids harboring either EPM1 or EPM2 mutation (Fig. 4C), although the expression levels of RFX1 were upregulated to a similar level under such conditions (Fig. 4D). This suggested that the effect of doxorubicin on HBV replication was specifically dependent on the EP element. Consistently, this phenomenon was also found in 1.2 × HBV EPM1‐ or EPM2‐mutant expression plasmid‐transfected HuH7 cells after receiving doxorubicin treatment (Fig. S6).

### The two conserved sequences of EP element in genotypes A–D HBV genomes both mediate doxorubicin‐induced promotion of HBV replication

**Figure 5:**
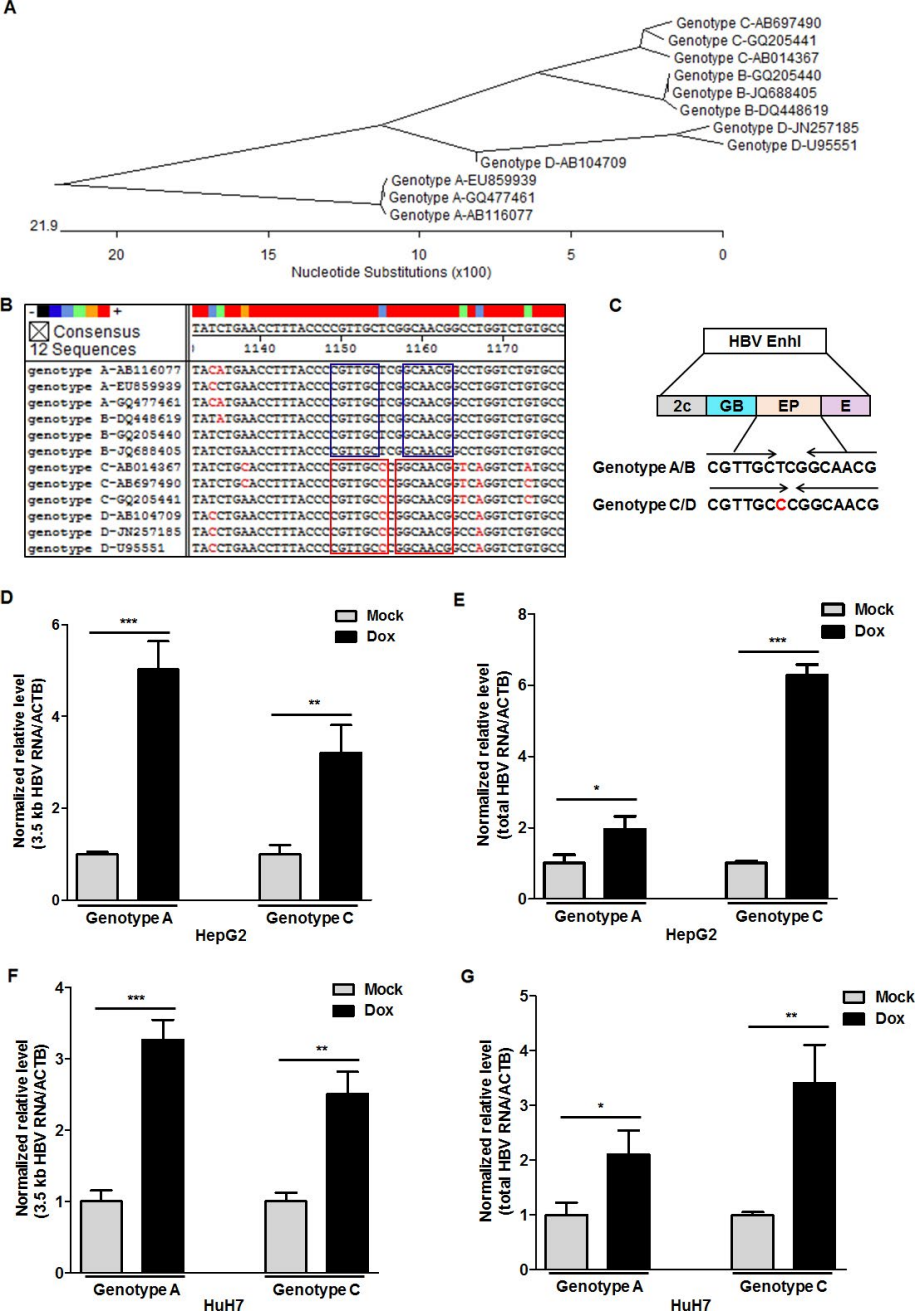
Doxorubicin promotes the replication of HBV harboring either of two conserved EP elements. (A) The reference sequences of the twelve HBV genomes of genotypes A–D were analyzed by a phylogenetic tree analysis. The accession numbers of the twelve HBV genomes were as follows: genotype A: EU859939, GQ477461, and AB116077; genotype B: GQ205440, JQ688405, and DQ448619; genotype C: AB697490, GQ205441, and AB014367; genotype D: JN257185, U95551, and AB104709. (B) The sequences of the EP element located in enhancer I of the different HBV genotypes were analyzed by MegAlign software (DNASTAR, Madison, WI). (C) The palindromic sequences of the two conserved EP elements among HBV genome genotypes A–D. HepG2 cells were transfected with 2 *μ*g pGEM‐1.3 × HBV expression plasmid (genotype A) or pBB4.5‐1.2 × HBV expression plasmid (genotype C) using lipofectamine 2000 in a 35‐mm dish. After 24 h of transfection, the cells were treated with 1 *μ*mol/L Dox for 1 h. The relative levels of 3.5 kb HBV RNA (D) and total HBV RNA (E) in cells were quantified by qRT‐PCR at 48 h post‐Dox treatment. ACTB mRNA was used as an internal control. According to the above treatment, the relative levels of 3.5 kb HBV RNA (F) and total HBV RNA (G) in HuH7 cells were quantified by qRT‐PCR.

Considering the important role of the EP element in the doxorubicin‐induced promotion of HBV replication, the sequences of the EP element were analyzed in the genomes of HBV genotypes A–D, which are the predominant genotypes worldwide (Fig. 5A). Two highly conserved sequences of the EP element in the genomes of HBV genotypes A–D were identified as follows: “CGTTGCTCGGCAACG” in the genomes of HBV genotypes A and B and “CGTTGCGCGGCAACG”, in which a single‐nucleotide “T” was changed to “G” in the genomes of HBV genotypes C and D (Fig. 5B). As shown in Figure 5C, different palindromic sequences were formed between two conserved sequences of the EP element due to a single‐nucleotide exchange. For this phenomenon, the effect of doxorubicin in HBV replication was detected in two representative genotypes: A and C. The results revealed that both the 3.5 kb HBV RNA and the total HBV RNA of either genotype A or C were upregulated in HepG2 cells treated with doxorubicin (Fig. 5D and E). Consistently, doxorubicin could promote the replication of HBV genotypes A and C in HuH7 cells (Fig. 5F and G).

## Discussion

Doxorubicin is a cytotoxic anthracycline antibiotic that was isolated from cultures of *Streptomyces peucetius* subsp. *Caesius* (ATCC 27952) and is used in chemotherapy treatment for various human cancers 47. Intercalation and enzyme inhibitions are two different mechanisms by which doxorubicin exerts its antitumor activity and ultimately leads to cell death through DNA disruption 48, 49. The cytotoxic anticancer drugs represented by doxorubicin are some of the drugs first reported to cause HBV reactivation 18, 50. Although the management of patients with hepatitis B infection receiving chemotherapy has been suggested in international guidelines, and nucleos(t)ide analogue (NA) treatment has proved to be effective in preventing HBV reactivation 51, 52, 53, new preventive approaches are still needed to be explored.

**Figure 6:**
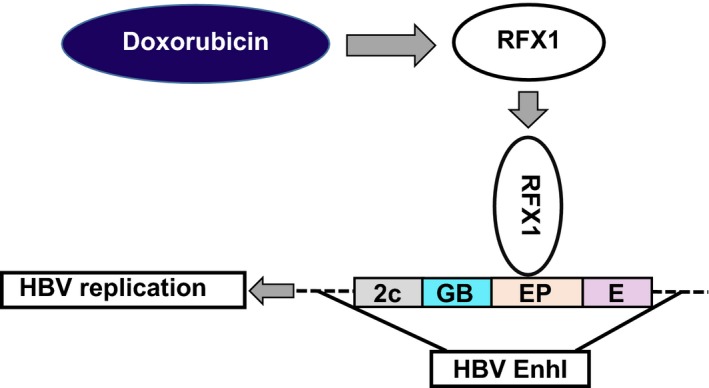
The pathway of RFX1 participating in the doxorubicin‐induced direct promotion of HBV replication. Doxorubicin promotes RFX1 expression and increases the amount of RFX1 bound to the EP element of HBV enhancer I. Subsequently, the activity of HBV enhancer I is enhanced, and HBV replication is promoted.

To elucidate the mechanism of cytotoxic chemotherapy‐induced HBV reactivation, the effect of doxorubicin and epirubicin, the representative cytotoxic anticancer drugs, in HBV replication, was analyzed. In line with previous reports, we found that doxorubicin and epirubicin could promote the expression of all HBV products, such as protein products consisting of HBsAg, HBeAg, HBc, and HBx, as well as nucleic acid products consisting of HBV DNA, 3.5 kb HBV RNA, and total HBV RNA. As we know, various HBV products are regulated by different regulatory sequences, including four promoters, that is, the preS1 promoter, preS2 promoter, X promoter, and core promoter, as well as two enhancers (enhancer I and II). In addition, two enhancers could regulate the activity of four promoters 54. Therefore, the current data demonstrated that doxorubicin promotes HBV replication by enhancing the activity of all four promoters. Interestingly, under doxorubicin treatment, the expression of RFX1, a transactivator of HBV replication, also increased in a dose‐dependent manner. The direct promotion of HBV replication induced by doxorubicin was significantly attenuated when endogenous RFX1 was knocked down by RFX1‐specific RFX1 shRNA. Further, we confirmed that doxorubicin could significantly increase the amount of RFX1 bound to HBV enhancer I. Moreover, doxorubicin‐promoted HBV replication was significantly reduced when the EP element located in the HBV enhancer I was mutated, suggesting that the participation of RFX1 in the promotion of HBV replication induced by doxorubicin was partially mediated by the EP element of HBV enhancer I. In addition, two conserved EP elements were found in the genome of HBV genotypes A‐D, both of which could lead to the promotion of HBV replication mediated by RFX1 under doxorubicin treatment. The above results suggested a pathway by which RFX1 participates in the doxorubicin‐induced direct promotion of HBV replication, in which doxorubicin promotes RFX1 expression and increases the amount of RFX1 bound to the EP element of HBV enhancer I, subsequently enhances the activity of HBV enhancer I and promotes HBV replication (Fig. 6).

As PML promotes HBV pgRNA expression by enhancing the activity of the basal core promoter in PML‐NB, it mediates the promotion of HBc and HBV DNA expressions induced by doxorubicin but not of HBsAg and HBeAg, while RFX1 mediates the doxorubicin‐induced promotion of all HBV products by enhancing the activity of the HBV enhancer I. Therefore, PML and RFX1 regulate HBV replication by acting on the different regulatory sequences and may synergistically participate in the direct promotion of HBV replication mediated by cytotoxic chemotherapy. However, there are some limitations in this study. (1) Although the cytotoxic chemotherapy‐mediated direct promotion of HBV replication may accelerate HBV reactivation, hepatitis relapse occurred during the recovery of immune response after HBV replication. Therefore, the inhibitory effect of doxorubicin in the immune system should be the main factor of hepatitis relapse, and it is difficult to address HBV‐related hepatitis in the present study. (2) Compared to rituximab, steroids, and other immunosuppressive agents, the incidence of HBV reactivation was relatively low in patients receiving chemotherapy with doxorubicin; thus, this study may only be helpful for some patients with HBV reactivation.

In conclusion, we confirmed that RFX1 could participate in the promotion of HBV replication induced by doxorubicin, a representative cytotoxic anticancer compound, and provided an alternative molecular mechanism by which doxorubicin could induce HBV reactivation in this study.

## Conflict of Interest

The authors have declared that no competing interest exists.

## Supporting information


**Table S1.** Summary of primer sequences for constructing gene expression plasmid.
**Table S2.** Summary of primers sequences for qPCR.
**Figure S1.** Doxorubicin promotes HBV replication in HepAD38 cells.
**Figure S2.** Doxorubicin promotes RFX1 expression and HBV replication in a dose‐dependent manner.
**Figure S3.** Doxorubicin promotes HBsAg level in the culture supernatant of PHH cells.
**Figure S4.** The ectopic expression of RFX1 was confirmed by Western Blot.
**Figure S5.** The cytotoxic effect of RFX1 in doxorubicin treated HepG2 cells was analyzed by CCK8 assay.
**Figure S6.** The role of EP element in RFX1‐mediated promotion of HBV replication in HuH7 cells.
