# Supplementary material for: RFX1 participates in doxorubicin‐induced hepatitis B virus reactivation
Source: Cancer Med. 2018 Mar 30;7(5):2021–33. doi: 10.1002/cam4.1468 (PMC5943424; doi:10.1002/cam4.1468)
Supplement: Supplementary file 1 — Table S1. Summary of primer sequences for constructing gene expression plasmid. Table S2. Summary of primers sequences for qPCR. Figure S1. Doxorubicin promotes HBV replication in HepAD38 cells. Figure S2. Doxorubicin promotes RFX1 expression and HBV replication in a dose‐dependent manner. Figure S3. Doxorubicin promotes HBsAg level in the culture supernatant of PHH cells. Figure S4. The ectopic expression of RFX1 was confirmed by Western Blot. Figure S5. The cytotoxic effect of RFX1 in doxorubicin treated HepG2 cells was analyzed by CCK8 assay. Figure S6. The role of EP element in RFX1‐mediated promotion of HBV replication in HuH7 cells. [file CAM4-7-2021-s001.docx]

**RFX1 participates in doxorubicin-induced hepatitis B virus reactivation**

Jie Wang^1^, Junqiao Jia^1^, Ran Chen^1^, Shanlong Ding^1^, Qiang Xu^1^, Ting Zhang^1^, Xiangmei Chen^1^, Shuang Liu^2*^, Fengmin Lu^1*^

**Supplementary data**

**Table S1. Summary of primer sequences for constructing gene expression plasmid**

| **Plasmid name** | **Sequences** |
| --- | --- |
| pCMV-HA-RFX1 | Forward:5’-CCGAATTCCCATGGCAACACAGGCGTATACTGA-3’  Reverse:5’-CCGCGGCCGCTTAGCTGGAGGGCAGCGCCTG-3’ |
| pBB4.5-HBV1.2 EPM1 | Forward:5’-CTGAACCTTTACCCCGGCCTATATGGCCGGTCAGGTCTCTGCC-3’  Reverse:5’-GGCAGAGACCTGACCGGCCATATAGGCCGGGGTAAAGGTTCAG-3’ |
| pBB4.5-HBV1.2 EPM2 | Forward:5’-CCTTTACCCCGTTGCCCATCCGATGGGGCAACGGTCAGGTCTC-3’  Reverse:5’-GAGACCTGACCGTTGCCCCATCGGATGGGCAACGGGGTAAAGG-3’ |
| pGL3-HBV EnhI-luciferase | Forward:5’-CTCGGTACCGCCTTTCTGTGTAAACAATATCTGA-3’  Reverse:5’-AGACTCGAGAAGGTTCCACGCATGCGG-3’ |
| pGL3-HBV EnhI (EPM1)-luciferase | Forward:5’-CTGAACCTTTACCCCGGCCTATATGGCCGGTCAGGTCTCTGCC-3’  Reverse:5’-GGCAGAGACCTGACCGGCCATATAGGCCGGGGTAAAGGTTCAG-3’ |
| pGL3-HBV EnhI (EPM2)-luciferase | Forward:5’-CCTTTACCCCGTTGCCCATCCGATGGGGCAACGGTCAGGTCTC-3’  Reverse:5’-GAGACCTGACCGTTGCCCCATCGGATGGGCAACGGGGTAAAGG-3’ |
| pRNA-U6.1-RFX1 shRNA | Forward:5’-GATCCGCCTCTACTGCCACTACTTATTCAAGAGATAAGTAGTGGCAGTAGAGGTTTTTTA-3’  Reverse:5’-AGCTTAAAAAACCTCTACTGCCACTACTTATCTCTTGAATAAGTAGTGGCAGTAGAGGCG-3’ |
| pRNA-U6.1-scramble shRNA | Forward:5’-GATCCGCCTGTCATCCGATCCATTATTCAAGAGATAATGGATCGGATGACAGGTTTTTTA-3’  Reverse:5’-AGCTTAAAAAACCTGTCATCCGATCCATTATCTCTTGAATAATGGATCGGATGACAGGCG-3’ |

**Table S2. Summary of primers sequences for qPCR**

| **Target mRNA** | **Forward 5’-3’** | | **Reverse 5’-3’** |
| --- | --- | --- | --- |
| RFX1 | | GTGATCCAAGGCGGCTACAT | CTCAGCCGTCTCATAGTTGTCC |
| 3.5kb HBV RNA | | CCTACTGTTCAAGCCTCCAAGC | CAGAGGCGGTGTCAAGGAGAT |
| total HBV RNA | | TGTGCCTTCTCATCTGCCG | GCCTCAAGGTCGGTCGTTGAC |
| CTBP | | TTCACCGTCAAGCAGATGAGAC | CTGGCTAAAGCTGAAGGGTTCC |
| ACTB | | CTACAGCTTCACCACCACGG | TCAGGCAGCTCGTAGCTCTTC |


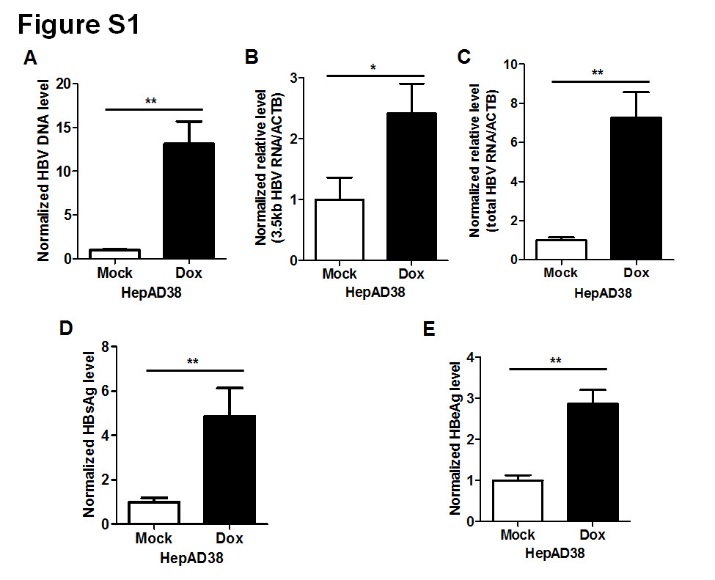


**Figure S1. Doxorubicin promotes HBV replication in HepAD38 cells.** The levels of HBV DNA (A), 3.5 kb HBV RNA (B) and total HBV RNA (C) in the culture supernatant of HepAD38 cells treated with 1 μM doxorubicin (Dox) for 1 hr were quantified by qRT-PCR at 48 hrs post Dox treatment. The levels of HBsAg (D) and HBeAg (E) in the culture supernatant of Dox treated HepAD38 cells were measured by a time-resolved fluoroimmunoassay.


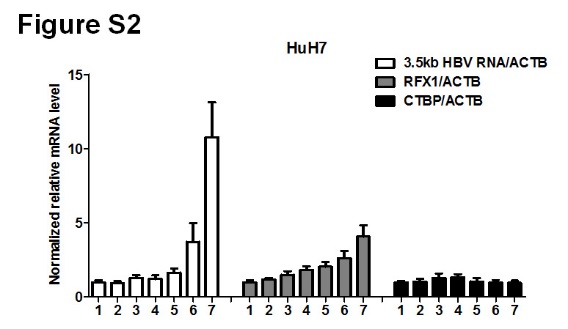


**Figure S2. Doxorubicin promotes RFX1 expression and HBV replication in a dose-dependent manner.** The pBB4.5-1.2×HBV expression plasmid (2 μg) was transfected into HuH7 cells using lipofectamine 2000 in a 6 cm dish. Then the transfected HepG2 cells were treated with a serial dosage of Dox for 1 hr. The relative levels of 3.5 kb HBV RNA, RFX1 and CTBP mRNA in cells were quantified by qRT-PCR at 48 hrs post Dox treatment. ACTB was used as an internal control. The final concentrations of doxorubicin were 1: 0 μM, 2: 0.05 μM, 3: 0.1μM, 4: 0.2 μM, 5: 0.5 μM, 6: 1 μM, 7: 2 μM.


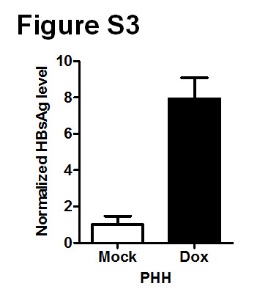


**Figure S3. Doxorubicin promotes HBsAg level in the culture supernatant of PHH cells.** 1×10^5^ primary human hepatocytes (PHH) cells in a 48-well plate were inoculated with 1×10^7^ copies of genome equivalent HBV in the presence of 4% PEG 8000 for 20 hrs. PHH cells were then washed with phosphate buffered solution (PBS) 6 times and maintained in PMM medium for 3 days. Then the HBV infected PHH cells were treated with 1 μM Dox for 1 hr. The levels of HBsAg in the culture supernatant of PHH cells were were measured by a time-resolved fluoroimmunoassay at 48 hrs post Dox treatment.


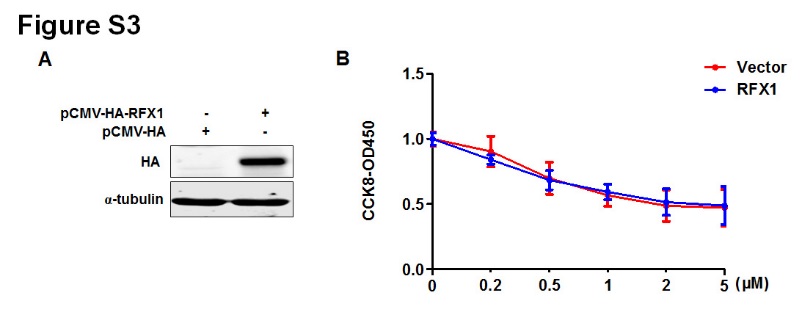


**Figure S4. The ectopic expression of RFX1 was confirmed by Western Blot.** HepG2 cells were transfected with 2 μg RFX1 expression plasmid (pCMV-HA-RFX1) or vector control (pCMV-HA) using lipofectamine 2000 in a 35 mm dish. The level of RFX1 protein was detected by Western Blot at 48 hrs post transfection.


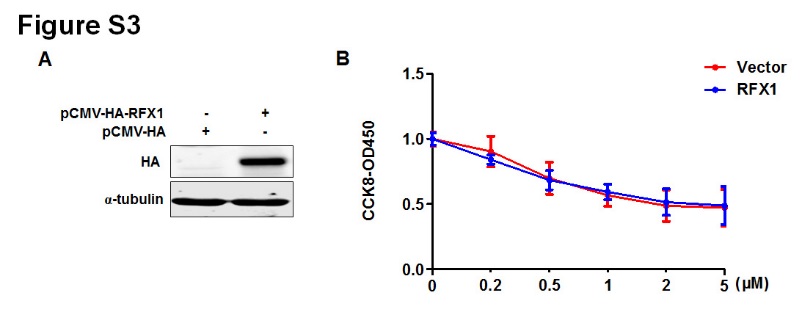


**Figure S5. The cytotoxic effect of RFX1 in doxorubicin treated HepG2 cells was analyzed by CCK8 assay.** HepG2 cells were treated with serial concentrations (0, 0.2, 0.5, 1, 2 and 5μM) of Dox for 1 hr. CCK-8 assays were used to detect cell viability at 48 hrs post Dox treatment. Data are shown as the mean ± standard deviation of 5 repeats in three independent experiments.


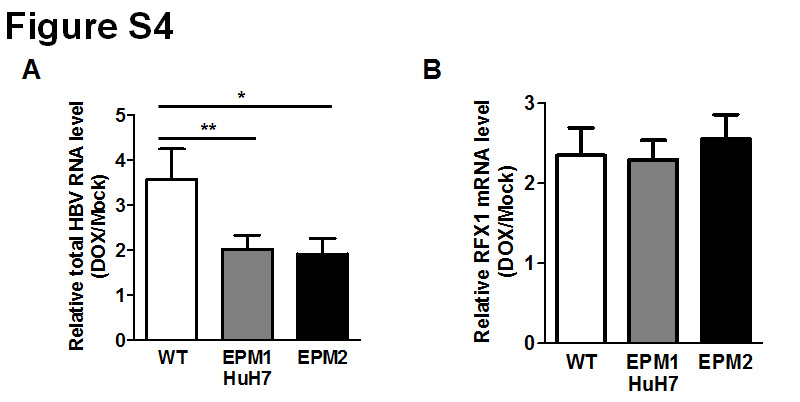


**Figure S6. The role of EP element in RFX1-mediated promotion of HBV replication in HuH7 cells.** HuH7 cells were transfected with wild-type, EPM1 or EPM2 mutant 1.2×HBV expression plasmid. After 24 hrs of transfection, the cells were treated with Dox for 1 hr. The levels of total HBV RNA (A) and RFX1 mRNA (B) were detected by qRT-PCR at 48 hrs post Dox treatment.
